# Influence of Age on Speech Recognition in Noise and Hearing Effort in Listeners with Age-Related Hearing Loss

**DOI:** 10.3390/jcm12196133

**Published:** 2023-09-22

**Authors:** Torsten Rahne, Telse M. Wagner, Anna C. Kopsch, Stefan K. Plontke, Luise Wagner

**Affiliations:** Department of Otorhinolaryngology, University Medicine Halle, Ernst-Grube-Straße 40, 06120 Halle (Saale), Germany; telse.wagner@gmail.com (T.M.W.);

**Keywords:** hearing in noise, age-related hearing loss, reference values, hearing effort, speech recognition

## Abstract

The aim of this study was to measure how age affects the speech recognition threshold (SRT_50_) of the Oldenburg Sentence Test (OLSA) and the listening effort at the corresponding signal-to-noise ratio (SNR_cut_). The study also investigated the effect of the spatial configuration of sound sources and noise signals on SRT_50_ and SNR_cut_. To achieve this goal, the study used olnoise and icra5 noise presented from one or more spatial locations from the front and back. Ninety-nine participants with age-related hearing loss in the 18–80 years age range, specifically in the 18–30, 31–40, 41–50, 51–60, 61–70, and 71–80 age groups, participated in this study. Speech recognition and listening effort in noise were measured and compared between the different age groups, different spatial sound configurations and noise signals. Speech recognition in noise decreased with age and became significant from the age group of 50–51. The decrease in SRT_50_ with age was greater for icra5 noise than for olnoise. For all age groups, SRT_50_ and SNR_cut_ were better for icra5 noise than for olnoise. The measured age-related reference data for SRT_50_ and SNR_cut_ can be used in further studies in listeners with age-related hearing loss and hearing aid or implant users.

## 1. Introduction

Human hearing typically deteriorates with age. Regarding the correlation of the age-related decline in outer hair cells and later in inner hair cells, the pure-tone hearing thresholds are negatively affected. In addition to pure-tone hearing loss, speech recognition thresholds in noise increase with age and may be correlated with reduced outer hair cell function [1]. Even individuals with normal pure-tone hearing thresholds (i.e., normal hearing listeners) may experience reduced speech recognition in noise with age [2], which may be caused by cognitive decline. Both peripheral dysfunction and cognitive factors contribute to age-related hearing loss and communication deficits, and are difficult to disentangle.

To assess speech recognition in noisy environments in relation to age, the HÖRSTAT study examined 1903 adults using the Göttingen Sentence Test (GÖSA) and pure-tone audiometry. The study found a gradual decline in pure-tone and speech recognition thresholds (SRTs) as age increases [2]. As part of the UK Biobank, around 500,000 individuals aged 40–69 years were evaluated for speech-in-noise hearing and cognition. The data show that speech-in-noise hearing decreases exponentially with age after the age of approximately 50. This contrasts with earlier audiogram data, which indicate a more linear decline in men under 40 years and consistently less hearing loss in women [3].

The decline in the ability to understand speech in older listeners cannot be solely attributed to the effects of aging on the auditory periphery and cognition [4,5]. Furthermore, cognitive abilities play a significant role in understanding speech in noisy settings [6,7]. It is important to take into account the contributions of central auditory processing at the brainstem and cortical levels. According to the hypothesis, age-related alterations in the balance between inhibitory and excitatory neural mechanisms modify the production of gamma oscillations, thereby influencing perceptual binding [8]. Consequently, speech comprehension in noisy environments is also affected. Among older adults, central auditory processing at the level of sensation, as indicated by sensory gating, has a minimal effect on speech recognition in noise. However, it significantly impacts perceptual organization [8].

Although hearing loss and age have been identified as the main contributors to decreased speech recognition in noisy environments [9], hearing in noise deficits could also start from an early age. There are also non-speech psychoacoustic aspects to consider, like dichotic signal detection, multi-burst masking, stream segregation, and modulation detection influencing speech recognition in noise, even when hearing aids are employed [10]. In a study carried out on young and elderly people with normal hearing conditions, Füllgrabe and Moore [11] proved that there is a strong correlation between the understanding of speech in noisy environments and sensitivity to temporal fine structures.

Speech perception in noise can be assessed using matrix sentence tests [12]. The Oldenburg Sentence Test (OLSA, HoerTech, Oldenburg, Germany) is a commonly used matrix test available for several languages, and it is accessible for clinical use [12,13,14]. It enables the evaluation of speech intelligibility in noisy scenarios for people with different levels of hearing loss. It includes a variety of noise signals that can be utilised to generate complex listening conditions with multiple sources of speech and noise. Various noise signals are utilised in clinical practice and research to measure speech reception thresholds (SRTs). Noise that has the same spectrum as speech (e.g., the olnoise employed in the OLSA) can provide effective masking. The SRT measured in fluctuating noise can be higher than that measured in stationary noise [15]. Weißgerber et al. [16] conducted a study on amplitude-modulated Fastl noise and assessed the speech recognition of a group of bimodal cochlear implant users, comparing their results to age-matched hearing aid users, individuals without subjective hearing loss, and a young normal hearing control group. The researchers observed that speech recognition in modulated noise was more considerably impacted than in continuous noise with an increase in hearing loss. In this study, olnoise generated by a male speaker and icra5 noise, which simulates speech with pauses, were used. The icra5 noise was originally developed using live English speech from the EUROM database by the International Collegium of Rehabilitative Audiology (ICRA) [17].

In clinical practice, speech recognition in noisy situations is measured using various parameters. The number and spatial positioning of speech and noise sources, as well as the type of speech material and adaptive procedures used, can all vary between measurements of speech recognition in noise [18,19,20]. This makes it difficult to compare results, due to the absence of reference data for individuals with normal age-related hearing across different age groups. For the first time, Decambron et al.’s study [21] has presented objective signal-to-noise ratio (SNR) values for varied age cohorts. The study assessed the 50% speech recognition threshold (SRT_50_) in noise via the ‘Vocale Rapide dans le Bruit’ test among 200 patients. The exam incorporated five noise sources with frontal speech presentation. The median values for SRT ranged from −0.37 dB (SNR) for those aged between 20–30 years to 6.84 dB (SNR) for individuals above 70 years of age. Mukari et al. [22] conducted a study on speech recognition in noisy environments using the Hearing in Noise Test (HINT). The researchers compared the Speech Reception Threshold (SRT) values obtained from three different noise sources: frontal, right and left. The study found evidence of an age-related decline in speech recognition ability, with older adults demonstrating poorer performance. Additionally, the research indicates that hearing thresholds have a significant impact on speech recognition ability in quiet conditions for older adults. However, it indicates that additional factors, including central auditory processing and cognitive abilities, could be more significant determinants in speech recognition performance in noisy environments.

In addition to assessing speech perception in noisy conditions, it is also important to evaluate listening effort. Active listening requires cognitive resources such as focus and attention. Decreased speech levels in quiet environments or the Signal-to-Noise Ratio (SNR) in noisy situations increase the demand on cognitive resources [23]. It is anticipated that age-related drops in pure-tone thresholds or hearing deficits beyond will lead to heightened listening effort, which may subsequently impact numerous daily communication scenarios with greater demands [23]. Listening effort increases with age regardless of hearing sensitivity [24]. According to the study by Kwak and Han [25], older adults experience a greater amount of listening effort compared to younger people as a result of background noise, directionality and ageing. A direct correlation was found between participants’ listening effort and their working memory and processing speed performance whilst understanding speech in background noise. Thus, it can be inferred that older adults require more cognitive resources to comprehend speech in such conditions [26].

Listening effort can be measured objectively by pupillometry. However, it was observed that pupil dilatation was less affected by SNR variation than subjective measures of listening effort [27]. The Adaptive Categorical Listening Effort Scaling Test (ACALES) is a subjective clinical procedure that has recently emerged offering an excellent means of measuring listening effort in noisy environments [24]. The sentential stimuli are presented alongside varying levels of background noise, quantified by signal-to-noise ratios (SNRs). The evaluation of listening effort is based on subjective responses and utilises either a 7-point or 14-point categorical scale that spans from a ‘no effort’ response to one indicating ‘extreme effort’. The SNRs of ACALES are adjusted presentation-by-presentation, based on prior subjective ratings. This guarantees comprehensive coverage across all possible categories and ultimately determines the moderate-level listening effort SNR_cut_ [26,28]. ACALES has been effectively employed to measure listening effort in individuals with hearing implants within a challenging acoustic environment [18,20].

This study aimed to evaluate the influence of age on the SRT_50_ of the Oldenburg sentence test (OLSA). For the first time, reference values for different age groups will be provided, including different spatial speech and noise configurations as well as noise signals. Noise will be presented in front (S_0_N_0_; reference and training condition) or behind (S_0_N_180_), as well as in a semicircle in front (S_0_N_0,45,−45_) or behind (S_0_N_135,180,−135_) of the participant. The study also examined for the first time how age affects listening effort when hearing in noisy environments.

## 2. Materials and Methods

This study consisted of exploratory cohorts and was prospective and non-interventional. It focused on adult volunteers with age- and sex-related pure-tone thresholds. The subjects were recruited in Halle (Saale), Germany, through personal contacts of the authors. The inclusion criteria required age-related hearing in both ears, fluency in German (native speakers), and to be between 18 and 80 years of age. Age-related hearing was confirmed when the bilateral pure-tone thresholds for air-conduction, averaged over 0.5, 1, 2, and 4 kHz (4PTA), were not worse than the age- and sex-related 95th percentile of the ISO 7026 [29] and there was symmetry between both ears. The Freiburg Monosyllables Test was used to measure the Word Recognition Score (WRS) of both ears, at a sound pressure level of 65 dB via headphones. Audiological evaluations were performed using an AT100 audiometer (Auritec, Hamburg, Germany).

Participants were excluded if they were unable to visit the study site, lacked fluency in the German language, demonstrated a lack of comprehension of the study procedures (also to ensure mental health), were pregnant, or did not meet the inclusion criteria. Informed written consent was obtained from all participants for their participation in the study. The study was conducted at the Audiology Lab of University Medicine Halle, Germany, after receiving ethical approval from the Medical Faculty of the Martin Luther University Halle-Wittenberg (approval number 2021-044) and was conducted in compliance with the Declaration of Helsinki.

The participants were selected from six age categories (18–30, 31–40, 41–50, 51–60, 61–70, 71–80 years). The sample size was calculated assuming an alpha of 0.05 and a power of 80%. The slope of the speech discrimination function at the reflection point, i.e., the SRT_50_, was about 17% per dB [13]. For sample size estimation in this study, a difference of at least 2 dB (SD = 2 dB) was considered necessary to account for a relevant SRT_50_ difference. Therefore, each age required at least 16 participants in the sample size.

**Figure 1:**
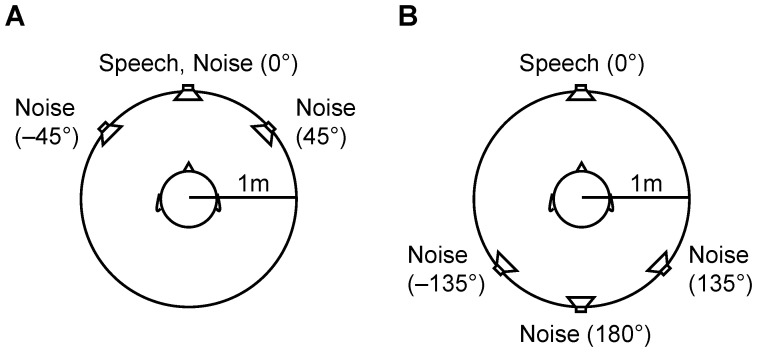
Experimental setup for speech and noise presentation. Speech signals were presented frontally, while noise signals were presented in a frontal (**A**) or backward (**B**) configuration.

For testing speech recognition in noisy environments, the German Matrix Sentence Test OLSA (Hörtech, Oldenburg, Germany) was used, which was played through four loudspeakers. Signal generation and presentation were conducted using the Oldenburger Measurement Application 2.2 R&D software from Hörtech in Oldenburg, Germany, along with a Gigaport eX audio interface from ESI Audiotechnik in Leonberg, Germany, and a PLMRA400 amplifier from Pyle in Brooklyn, NY, USA. Continuous noise signals were presented at a sound pressure level of 65 dB. The stimuli utilised were either the generic noise of the Oldenburg Logatome Speech Corpus with a male voice (olnoise) or the icra5 noise. Lists of 20 sentences were presented in front (S_0_N_0_; reference and training condition) or behind (S_0_N_180_), as well as in a semicircle in front (S_0_N_0,45,−45_) or behind (S_0_N_135,180,−135_), of the participant, all at a distance of 1 metre from the head centre (refer to Figure 1). In clinical practice, many different spatial configurations of speech and noise sources are used. Since many hearing aid or cochlear implant algorithms aim for a better understanding of speech coming from the front and to mitigate noise coming from frontal and other directions, we focus on speech coming from the front in this study. This is also relevant for the elderly who are to be included in this study. For each sentence, the sound pressure level was adjusted based on the participant’s response to the preceding sentence. This technique was performed to measure the open-set speech recognition threshold (SRT) for 50% correct recognition (SRT_50_), which was the primary endpoint for the S_0_N_0_ condition using olnoise, as well as a secondary endpoint for all other spatial conditions and the icra5 noise.

Listening effort was assessed utilizing ACALES v2.2 software (Hörtech, Oldenburg, Germany). The participants received a sequence of two consecutive sentences from the OLSA, with diverse signal-to-noise ratios (SNRs), presented frontally, in a background noise continuously played at 65 dB SPL (either olnoise or icra5) either from the frontal (S_0_N_0_) or rear (S_0_N_180_) loudspeaker after two training runs. Following the presentation of two OLSA sentences in noise, participants were instructed to rate their listening effort on a scale of eight response categories, ranging from ‘no effort’ to ‘only noise’. The signal-to-noise ratio (SNR) was changed adaptively for every round of two sentences, based on the previous assessment of the subjectively perceived listening effort, and was presented at a different SNR ranging between −40 dB and +20 dB. A listening effort function was computed by the software. The SNR_cut_, which represents the signal-to-noise ratio (SNR) at which a moderate effort was measured (called SNR_cut_), was assessed as a secondary endpoint for all experimental conditions by identifying the cutting point at 4 ESCU.

All the participants were seated and their heads were immobilised with a Papillon head fixation system (Focal Meditech, Tilburg, The Netherlands). After completing two OLSA and two ACALES training sessions, all participants accomplished 15 testing runs in which the noise signals and spatial conditions were applied in a pseudorandom sequence.

The primary and secondary endpoints were analysed descriptively in this exploratory study and normality was assessed using the Shapiro–Wilk test. An ANOVA for repeated measures was used to compare the distributions of SRT_50_ and SNR_cut_ across both the ‘noise signal’ and ‘spatial configuration’ experimental conditions, as well as the ‘age group’ factor among subjects. Mauchly’s test was used to verify the assumption of sphericity. The Bonferroni correction was employed to adjust degrees of freedom for all post hoc comparisons. Statistical analyses were conducted using version 28 of the SPSS software (IBM, Ehningen, Germany). Linear regression analysis was performed to measure the influence of the variables ‘age’ and ‘binaurally averaged PTA’ on the SRT_50_ and SNR_cut_.

## 3. Results

**Figure 2:**
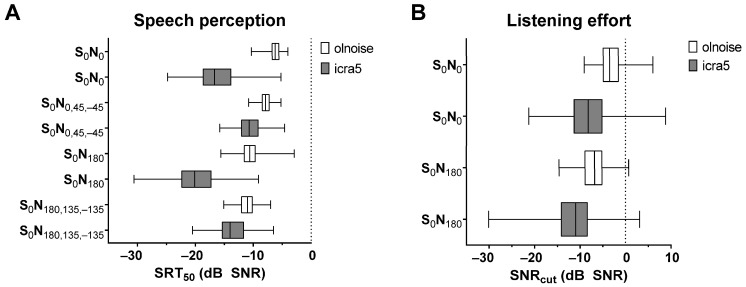
Speech recognition (**A**) and listening effort (**B**) in noise for all spatial conditions with olnoise (white) or icra5 noise (grey), presented as box plots (median, min, max), for all included participants.

All participants successfully completed the experiment. The demographic data of participants from all age groups are presented in Table 1. Figure 2 provides a comparison of SRT_50_ and SNR_cut_ across all spatial configurations of signal and noise sources for all participants. All spatial conditions demonstrated a better SRT_50_ and reduced listening effort for icra5 noise compared to olnoise.

**Figure 3:**
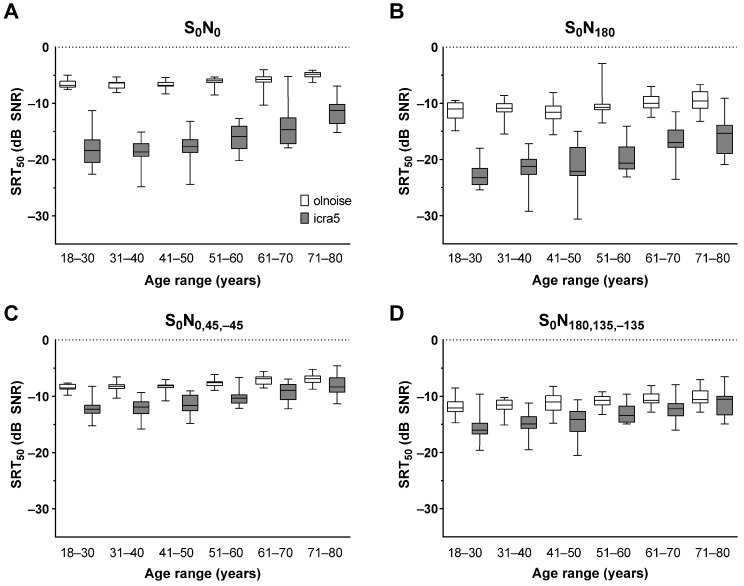
Speech recognition in noise as box plots (median, min, max) for all age groups for the spatial conditions of (**A**) S_0_N_0_, (**B**) S_0_N_180_, (**C**) S_0_N_0,45,−45_, and (**D**) S_0_N_180,135,−135_. The SRT_50_ is shown as box plots for the use of olnoise (white) or icra5 noise (grey).

The box plots in Figure 3 depict the SRT_50_ distributions across all age groups. Table 2 provides the specific means and standard deviations for all conditions. The ANOVA conducted on the SRT_50_ distribution demonstrated significant main effects of noise signal (F(1,89) = 1730.8, *p* < 0.001), spatial configuration (F(2.41,223.5) = 488.5, *p* < 0.001) and the between-subjects factor of age group (F(5,89) = 270.3, *p* < 0.001). Interactions between the noise signal and age group (F(5) = 18.3, *p* < 0.001), spatial configuration and age group (F(12.6) = 2.1, *p* < 0.01), noise signal and spatial configuration (F(2.43,216.6) = 738.9, *p* < 0.001) and noise signal and spatial configuration × age group (F(12.2) = 3.35, *p* < 0.001) were also significant. The post hoc comparison showed a better (lower) SRT_50_ for icra5 noise than for olnoise overall and in all age groups. The mean SRT_50_ was lowest (best) for the S_0_N_180_ presentation, significantly different between all spatial configurations with an increase to S_0_N_180,135,−135_, S_0_N_0_ and S_0_N_0,45,−45_. The post hoc comparison showed a significant increase between the age groups 18–50 and 61–80 years when olnoise was used. However, when icra5 noise was used, the difference was significant between the age groups 18–30 years, 51–80 years and between the age groups 31–50 years and 61–80 years.

**Figure 4:**
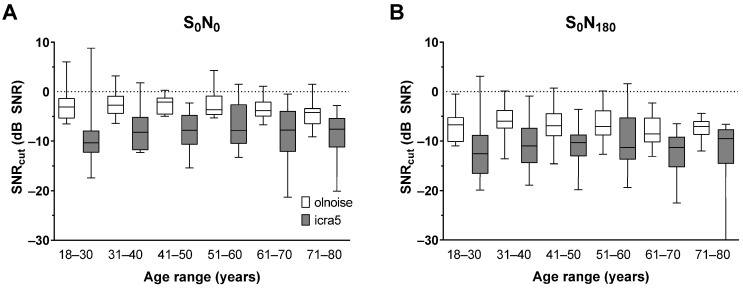
Listening effort in noise as box plots (median, min, max) for all age groups for the spatial conditions of (**A**) S_0_N_0_ and (**B**) S_0_N_180_. The SNR_5cut_ is shown as box plots for the use of olnoise (white) or icra5 noise (grey).

Figure 4 shows the SNR_cut_ distributions for all age distributions, used noise signals and spatial distributions. The specific means and standard deviations are summarised in Table 2. The ANOVA of the SNR_cut_ distribution revealed significant main effects of noise signal (F(1,92) = 266.9, *p* < 0.001), spatial configuration (F(1,92) = 228.2, *p* < 0.001), but not for the between-subjects factor of age group. Only the interaction between noise signal and spatial configuration was significant (F(1,92) = 8.12, *p* < 0.01). The post hoc comparison showed a lower SNR_cut_ for icra5 noise than for olnoise overall and for all spatial configurations. The mean SNR_cut_ was better for the S_0_N_180_ presentation compared to the S_0_N_0_ condition.

Table 3 lists the linear regression results for the variables of age and the binaural PTA. For the S_0_N_0_ condition with olnoise, a significant linear regression was observed between age and SRT_50,_ and age and SNR_cut_. The linear regression between age and speech perception in noise was also significant for the S_0_N_0,45,−45_ conditions, but not for all other conditions. Binaurally averaged PTA was correlated with speech perception in noise for all conditions except for the S_0_N_0_ condition. The linear regression between binaural PTA and listening effort in noise was not significant.

## 4. Discussion

The aim of this study was to provide age-related reference signal-to-noise ratios at which 50% speech recognition in noise (SRT_50_) and moderate listening effort (SNR_cut_) are assessed by participants. A decline in speech recognition in noise was observed with increasing age. This decrease was significant from the age group of 51–60 years and above. The decrease in SRT_50_ with age was greater when icra5 noise was used than when olnoise was used. Overall, speech could be discriminated at lower SNRs with icra5 noise than with olnoise. Since icra5 allows for listening into gaps in the noise signal (‘gap listening’ [30]), this finding is consistent with previous results using other speech-modulated noise signals [16,31,32]. For olnoise, the decrease from 18–30 to 71–80 years was smallest for the S_0_N_0,45,−45_ condition (1.6 dB) and largest for the S_0_N_180,135,−135_ condition (2.12 dB). Using icra5, this difference was also smallest for the S_0_N_0,45,−45_ condition (4.46 dB), but largest for the S_0_N_0_ condition (6.93 dB). The spatial configuration itself also influenced the age-related SRT_50_ and was observed in all age groups. When speech was presented from the front and noise from the back, the best SRT_50_ was observed for both noise signals used. This is consistent with spatial release from masking due to the separation of speech and noise sources [33,34]. However, the measured improvements are still smaller than in a similar study by Decambron et al. [21].

For olnoise, the SRT_50_ was −6.64 dB (SD: 0.75) for the 18–30-year-olds, which is within the range originally reported for the OLSA matrix test [13] and later research [14]. Adding more noise sources improved speech recognition in noise, as the spatial release of masking was increased by adding more correlated noise signal. However, in icra5, the addition of two more noise sources decreased speech intelligibility and thus worsened the SRT_50_. This finding may be due to the uncorrelated noise signals used in this condition.

Listening effort in noise was influenced by the noise signal used. Speech recognition in icra5 was rated as less demanding, resulting in a lower (better) SNR_cut_ compared to the olnoise conditions. Apparently, the gaps in the speech-modulated noise resulted in better speech recognition, which was rated as less demanding. The presentation of speech and noise from separate sources resulted in less listening effort. No age effect was observed for all spatial and noise conditions.

The observed standard deviations of the SNR_cut_ were larger than those of the SRT_50_. This may be due to the difficulty of the task. Although the ACALES task was explained in detail, it cannot be excluded that some participants focused more on the speech recognition rating than on the actual listening effort. In such cases, a different internal scale would have been used. The resolution of the scale, with only seven levels, may also have contributed to the larger standard deviation.

Age correlates with increased pure-tone thresholds. It is therefore difficult to disentangle the influence of both factors on speech perception and listening effort in noise. In this study, the influence of the binaurally averaged PTA on both endpoints was assessed by regression analysis. As expected, a correlation between this PTA and the SRT_50_ was observed for most conditions. For the clinically relevant condition of S_0_N_0_ presentation of noise and speech, this correlation was not significant, but the effect of age was. We conclude that both age and PTA may influence speech perception in noise, at least partially independently. However, the study design, especially the inclusion criteria, has limitations for such an analysis. At younger ages, only participants with good PTA thresholds were included, whereas participants with poorer PTA were only included at older ages. This is reflected in the larger standard deviations and percentiles in the older age groups. This means that younger participants with increased PTA were excluded from the study, which limits a sufficient analysis of the influence of PTA. Also, a specific analysis of high frequency hearing loss, which may correlate with speech-in-noise difficulties, was not performed in this study. Both pure-tone thresholds and age are correlated, but are not the only factors in speech recognition in noise that decline with age. However, many of these factors have to be taken into account when age-related reference values are provided for different age groups.

As speech recognition in noise and listening effort are measured in clinical routines with different spatial configurations and noise signals, this study provides reference data for normal hearing listeners of all ages. The reference values will be useful for comparisons with patient groups such as those with idiopathic sudden hearing loss [35]. In addition to speech recognition in quiet conditions, improved hearing in noise conditions is one of the goals of cochlear implantation and can be used as an indication criterion [36,37]. Especially in patients with unilateral deafness, hearing in noise can be significantly improved by a cochlear implant [38,39,40,41,42] and may be cost-effective compared to no intervention or other interventions [43]. A study of patients with different indications for speech recognition in quiet and noise conditions showed that those patients undergoing cochlear implant candidacy testing who qualified only by a poor hearing in noise had improved hearing in both quiet and noise conditions [44]. As these effects may also be caused by the pre-processing technology in cochlear implants, hearing aid or active middle-ear implant users, different spatial configurations will be required to demonstrate benefits. The age-related reference data reported here may also serve as a benchmark in these cases.

The study results are limited to the German language, as only German-speaking participants were included. In addition, other spatial configurations of speech and noise as well as different noise signals are not covered by the study results. The study did not specifically analyse frequency-specific pure-tone thresholds, which may have differed between age groups.

In conclusion, this study provides reference data on speech perception and listening effort in noise for listeners with age-related hearing loss. In addition to the clinically most relevant spatial condition with frontal speech and noise presentation, this study also provides reference data for more complex spatial conditions and different noise signals.

## Figures and Tables

**Table 1:** Demographic data.

				Age Group, Years					
Characteristics				18–30	31–40	41–50	51–60	61–70	71–80
Number				18	16	15	18	16	16
Age, mean (SD), years				24.6 (3.9)	34.5 (2.7)	46.8 (2.7)	53.0 (1.5)	66.2 (2.9)	75.8 (2.9)
Men/women, N				14/4	13/3	8/7	12/6	12/6	13/3
Hearing, mean (SD), dB PTA									
	Right ear			8.0 (4.7)	8.3 (3.6)	13.3 (5.2)	17.1 (7.4)	22.3 (9.5)	25.9 (8.8)
		Median [25th, 75th percentiles]		7.5 [5.0,10.0]	6.9 [6.3,11.3]	13.8 [10.0,17.5]	15.6 [11.3,20.0]	19.4 [15.0,29.7]	25.3 [19.4,34.1]
	Left ear			6.8 (5.1)	8.4 (4.5)	14.1 (5.1)	17.1 (6.4)	22.6 (8.2)	27.3 (11.2)
		Median [25th, 75th percentiles]		6.3 [3.8,7.5]	7.5 [5.0,12.2]	11.3 [10.0,18.8]	15.6 [12.2,22.8]	22.5 [13.1,28.8]	30.0 [17.8,36.9]
	Average over both ears			7.5 (5.3)	8.5 (3.8)	14.4 (4.5)	17.5 (6.8)	23.1 (8.4)	26.6 (9.7)
		Median [25th, 75th percentiles]		6.8 [4.4,9.4]	8.8 [5.6,11.3]	14.1 [10.5,18.3]	17.2 [12.5,20.8]	21.3 [15.0,30.0]	27.8 [18.6,35.6]
Word recognition, mean (SD), % correct @ 65dB SPL									
	Right ear			99.0 (2.1)	99.0 (2.1)	97.9 (3.2)	96.3 (4.3)	88.3 (14.4)	87.8 (8.2)
		Median [25th, 75th percentiles]		100 [100,100]	100 [100,100]	100 [95,100]	95 [95,100]	95 [80,100]	90 [81.3,93,8]
	Left ear			99.0 (2.8)	98.3 (3.1)	98.2 (2.5)	96.6 (5.7)	90.0 (11.6)	84.0 (13.8)
		Median [25th, 75th percentiles]		100 [100,100]	100 [95,100]	100 [95,100]	100 [95,100]	95 [80,100]	87.5 [72.5,95]

Abbreviations: SD: standard deviation; dB PTA_0.5-4_: Decibel pure tone average over 0.5, 1, 2, and 4 kHz; SPL: Sound pressure level.

**Table 2:** Outcome measures of speech perception and listening effort in noise.

			Age Group, Years					
Outcome			18–30	31–40	41–50	51–60	61–70	71–80
Speech perception in noise								
	SRT_50_ in dB SNR, mean (SD)							
		Olnoise, S_0_N_0_	−6.64 (0.75)	−6.53 (0.65)	−6.54 (0.58)	−6.18 (0.78)	−5.95 (1.38)	−4.91 (0.60)
		Olnoise, S_0_N_180_	−11.43 (1.58)	−11.11 (1.63)	−11.19 (1.62)	−10.46 (2.32)	−9.75 (1.48)	−9.54 (1.84)
		Olnoise, S_0_N_0,45,−45_	−8.51 (0.74)	−8.19 (0.85)	−8.14 (0.60)	−7.74 (0.71)	−7.10 (0.87)	−6.91 (0.91)
		Olnoise, S_0_N_180,135,−135_	−12.35 (1.58)	−11.57 (1.26)	−11.06 (1.47)	−11.04 (1.11)	−10.40 (1.38)	−10.23 (1.69)
		Icra5, S_0_N_0_	−18.53 (2.67)	−18.66 (2.27)	−17.39 (1.73)	−16.39 (2.28)	−14.11 (3.47)	−11.60 (2.37)
		Icra5, S_0_N_180_	−22.52 (2.34)	−21.41 (2.81)	−20.44 (3.05)	−19.98 (2.58)	−16.87 (3.10)	−15.78 (3.53)
		Icra5, S_0_N_0,45,−45_	−12.41 (1.27)	−11.89 (1.68)	−11.01 (1.52)	−1.13 (1.37)	−9.25 (1.78)	−7.95 (1.77)
		Icra5, S_0_N_180,135,−135_	−16.20 (1.74)	−14.67 (1.75)	−14.26 (1.95)	−13.34 (1.57)	−12.33 (2.05)	−11.20 (2.25)
Listening effort in noise								
	SNR_cut_ in dB SNR, mean (SD)							
		Olnoise, S_0_N_0_	−3.2 (2.4)	−2.5 (2.6)	−2.4 (1.8)	−2.5 (3.0)	−3.6 (2.1)	−4.6 (2.5)
		Olnoise, S_0_N_180_	−7.9 (2.6)	−5.8 (3.6)	−6.8 (3.6)	−6.8 (3.5)	−8.2 (3.1)	−7.6 (2.4)
		Icra5, S_0_N_0_	−10.3 (4.6)	−7.6 (4.1)	−7.9 (3.9)	−6.8 (5.1)	−9.1 (5.4)	−8.7 (4.4)
		Icra5, S_0_N_180_	−13.5 (4.4)	−10.4 (4.9)	−10.6 (3.7)	−10.7 (5.8)	−12.8 (4.5)	−12.0 (6.0)

Abbreviations: SD: standard deviation; SRT_50_: 50% speech reception threshold, SNR: signal-to-noise ration; SNR_cut_: signal to noise ratio at moderate listening effort.

**Table 3:** Linear regression report for the outcome variables.

	Dependent Variable	Age					PTA, Binaural Average				
Outcome		b	SE	β	T	*p*	b	SE	β	T	*p*
Speech perception in noise											
	Olnoise, S_0_N_0_	0.025	0.007	0.438	3.42	**<0.001**	0.013	0.014	0.124	0.97	0.335
	Olnoise, S_0_N_180_	0.006	0.015	0.055	0.40	0.689	0.087	0.027	0.441	3.23	**0.002**
	Olnoise, S_0_N_0,45,−45_	0.018	0.007	3.240	2.74	**0.007**	0.037	0.013	0.348	2.94	**0.004**
	Olnoise, S_0_N_180,135,−135_	0.010	0.011	0.114	0.88	0.384	0.069	0.021	0.421	3.22	**0.002**
	Icra5, S_0_N_0_	0.035	0.019	0.172	1.89	0.063	0.253	0.035	0.663	7.26	**<0.001**
	Icra5, S_0_N_180_	0.031	0.020	0.140	1.49	0.140	0.276	0.038	0.689	7.34	**<0.001**
	Icra5, S_0_N_0,45,−45_	0.042	0.012	0.341	3.43	**<0.001**	0.109	0.023	0.469	4.70	**<0.001**
	Icra5, S_0_N_180,135,−135_	0.026	0.015	0.176	1.70	0.093	0.161	0.028	0.591	5.70	**<0.001**
Listening effort in noise											
	Olnoise, S_0_N_0_	−0.044	0.022	−0.301	−2.04	**0.044**	0.026	0.041	0.094	0.64	0.524
	Olnoise, S_0_N_180_	−0.031	0.027	−0.175	−1.16	0.248	0.015	0.050	0.045	0.30	0.766
	Icra5, S_0_N_0_	−0.032	0.041	−0.117	−0.78	0.441	0.092	0.076	0.181	1.20	0.232
	Icra5, S_0_N_180_	−0.042	0.043	−0.149	−0.98	0.330	0.079	0.080	0.149	0.98	0.331

Bold: significant linear regression, *p* < 0.05.

## Data Availability

Supporting raw data may be obtained through special request from the corresponding author.
